# Secondary bacterial infections and antibiotic resistance among tungiasis patients in Western, Kenya

**DOI:** 10.1371/journal.pntd.0005901

**Published:** 2017-09-08

**Authors:** Ruth Monyenye Nyangacha, David Odongo, Florence Oyieke, Missiani Ochwoto, Richard Korir, Ronald Kiprotich Ngetich, Gladys Nginya, Olipher Makwaga, Christine Bii, Peter Mwitari, Festus Tolo

**Affiliations:** 1 Center for Traditional Medicine and Drug Research, Kenya Medical Research Institute, Nairobi, Kenya; 2 School of Biological Sciences, University of Nairobi, Nairobi, Kenya; 3 Production Department, Kenya Medical Research Institute, Nairobi, Kenya; 4 Center for Microbiology Research, Kenya Medical Research Institute, Nairobi, Kenya; 5 Center for Infectious and Parasitic Diseases Control Research, Kenya Medical Research Institute, Busia, Kenya; Mahidol University, THAILAND

## Abstract

Tungiasis or jigger infestation is a parasitic disease caused by the female sand flea *Tunga penetrans*. Secondary infection of the lesions caused by this flea is common in endemic communities. This study sought to shed light on the bacterial pathogens causing secondary infections in tungiasis lesions and their susceptibility profiles to commonly prescribed antibiotics. Participants were recruited with the help of Community Health Workers. Swabs were taken from lesions which showed signs of secondary infection. Identification of suspected bacteria colonies was done by colony morphology, Gram staining, and biochemical tests. The Kirby Bauer disc diffusion test was used to determine the drug susceptibility profiles. Out of 37 participants, from whom swabs were collected, specimen were positive in 29 and 8 had no growth. From these, 10 different strains of bacteria were isolated. Two were Gram positive bacteria and they were, *Staphylococcus epidermidis* (38.3%) and *Staphylococcus aureus* (21.3%). Eight were Gram negative namely *Enterobacter cloacae* (8.5%), *Proteus species* (8.5%), *Klebsiellla species* (6.4%), *Aeromonas sobria* (4.3%), *Citrobacter species* (4.3%), *Proteus mirabillis*(4.3%), *Enterobacter amnigenus* (2.1%) and *Klebsiella pneumoniae* (2.1%). The methicillin resistant *S*. *aureus* (MRSA) isolated were also resistant to clindamycin, kanamycin, erythromycin, nalidixic acid, trimethorprim sulfamethoxazole and tetracycline. All the Gram negative and Gram positive bacteria isolates were sensitive to gentamicin and norfloxacin drugs. Results from this study confirms the presence of resistant bacteria in tungiasis lesions hence highlighting the significance of secondary infection of the lesions in endemic communties. This therefore suggests that antimicrobial susceptibility testing may be considered to guide in identification of appropriate antibiotics and treatment therapy among tungiasis patients.

## Author summary

Secondary bacterial infection of tungiaisis lesions is a threat to jigger infested patients. Once the flea penetrates the skin, it leaves an opening on the skin through which it lays eggs, defecates and breathes throughout it’s life cycle on the host. At the same time, it feeds on the host’s blood hence a direct connection of the environment to the blood stream is established. Chances of bacteria getting into the blood stream is therefore greatly enhanced. Once bacteria gets into the blood, it can lead to life threatening conditions like septicemia, meningitis, pneumonia and toxic shock syndrome. Affected communities are oblivious to this danger and they neither seek nor get treated for this parasitosis. The health officials and scientific community also continue to ignore this disease hence it’s extremely neglected. Consequently, not much is understood concerning the disease dynamics, distribution and pathogenesis. This study therefore is a deliberate effort to bring attention to one aspect of the disease dynamics of this menace.

## Introduction

Tungiasis is a parasitic disease caused by the sand flea *Tunga penetrans* [1].The fleas can infest any part of the body. However majority of the cases occur on the feet [2].Children and the elderly bear the brunt of the infection in endemic areas [3], [4]. During the transmission period, a study that followed up individuals entering an endemic area was able to demonstrate that by the third week all the participants were infested by *T*. *penetrans* [5]. The ectoparasites can cause more than 50 lesions in a single individual in some cases [1]. This leads to severe inflammation and ulceration which is associated with intense pain. Walking, working or going to school becomes a problem. In endemic communities it’s not uncommon to find children who have dropped out of school due to the pain and stigma brough about by this condition. In some cases there is loss of toe nails and deformation of digits [6].

Secondary infection of the lesions caused by *Tunga* species occurs in endemic areas. A bacteriological investigation of the lesions from human tungiasis in Brazil reported isolation of various pathogenic bacteria [7]. Untreated Tungiasis is a risk factor in acquiring blood stream bacterial infections (bacteremia) due to broken skin. Once the jigger flea penetrates the skin, it maintains an opening (250 to 500μm) in the epidermis through which it defecates, breathes and lays eggs, consequently connecting the outer surface of the skin and the blood stream as it feeds [7], [8]). The exposed skin tissue is a perfect environment for bacteria to thrive. It provides warmth, moisture and nutrients, factors that are essential for microbial growth [9].

Sepsis in Tungiasis patients has been elucidated hence illustrating the medical significance of systemic infections caused by secondary bacteria infection in these patients [10], [11]. Systemic conditions like pneumonia, meningitis, osteomyelitis, endocarditis, septicemia and Toxic shock syndrome (TSS) can be fatal [12].

Antibiotic resistance compounds the problem, as treatment options are greatly diminished due to bacteria developing mechanisms that neutralize available antibiotics [13]. The selection of appropriate antibiotics for treatment of severe tungiasis is critical for proper management of this parasitosis. This study therefore sought to shed light on the bacterial pathogens causing secondary infections in tungiasis lesions and their susceptibility profiles to commonly used antibiotics.

## Materials and methods

### Study area

The study area was Vihiga County in Western Kenya. It is one of the most densely populated rural areas in Kenya.The population density as of 2009, was 1,045 persons per square kilometre. This figure is projected to rise to 1231 persons per square kilometer in 2017. The poverty levels are consequently very high due to the population pressure on land and other resources. The GDP per capita income was reported as US $ 1,103 in 2013. Majority of the inhabitants own small uneconomical pieces of land as a result of increased subdivision occassioned by the high population and cultural practice of land inheritance.

The area has two rainy seasons. Long rainy season in April, May and June and the short rains in September, October and November. The study took place during the dry and hot season from January to March, 2016. Tungiasis peaks during the dry season.

### Bacteria isolation

Participants were recruited with the help of Community Health Workers. Swabs were taken from lesions which clinically showed signs of secondary infection like swelling, erythema and pus.

The flea was extracted with a sterile needle after disinfection of the surrounding skin with 70% alcohol for 1 minute [7]. Swabs were then collected from the surgical lesions by use of sterile cotton swabs. The cotton swabs were moistened in sterile physiological saline and gently moved in and out of the remaining cavity. One swab was used for each lesion and labeled accordingly. The swab was then transferred to a sterile transportation tube, briefly stored in an ice box and transported to the laboratory. Upon arrival at the laboratory, the swabs were cultured separately and directly onto Mannitol salt and MacConkey agar (both from Oxoid Ltd, Basingstoke, United Kingdom). They were also inoculated on Brain heart infusion agar (Oxoid Ltd, Basingstoke, United Kingdom) supplemented with the commercially available 5% sheep blood (TCS Biosciences, Botolph Claydon, Buckingham, United Kingdom) and incubated aerobically at 35°C for 24 hours.

Identification of suspected bacteria colonies was done by colony morphology, Gram staining, catalase, coagulase tests and biochemical tests as described previously [14]. Anaerobic bacteria were not isolated due to limited laboratory facilities at the time.

### Antimicrobial susceptibility testing

The bacterial isolates were subjected to antimicrobial sensitivity testing by disk diffusion method as described by [15]. Briefly, test organisms were suspended in sterile normal saline to conform to 0.5 McFarland turbidity standard. With the aid of sterile cotton swab the suspended organisms were spread on Mueller-Hinton (Oxoid Ltd, Basingstoke, United Kingdom) Agar plate and the antibiotic disks dispensed. The plates were incubated at 37°C for 16-18h. Inhibition zone diameters were determined and recorded in Excel sheets and interpreted according to the Clinical and Laboratory Standards Institute (CLSI) guidelines [16].

The following panel of antibiotics (all from Oxoid) and their concentrations were used. For Gram negatives cefuroxime sodium 30 μg, amoxycillin\clavulanic acid 2:1 30 μg, chloramphenicol 30 μg, tetracycline 30μg, co-trimoxazole sxt 25 μg, nalidixic acid 30 μg, ampicillin 10 μg, ceftazidime 30 μg, cefotaxime 30 μg, ciprofloxacin 5 μg, norfloxacin 10μg, gentamycin 10μg, were tested. For Gram positives meropenem 10 μg, gentamycin 10μg, kanamycin 30μg, clindamycin 2μg, norfloxacin 10μg, ofloxacin 5μg, oxacillin 5μg, erythromycin 10μg, nalidixic acid 30 μg, trimethoprim-sulfamethoxazole 25μg, chloramphenicol 30 μg, tetracycline 30μg were tested.

### Ethics statement

The study got approval from the KEMRI Scientific and Ethics Review Unit (SERU). Approval number KEMRI/SERU/CTMDR/015/3116. Informed written consent was obtained from all participants including children, where the guardian provided an informed consent on their behalf. All data analyzed was coded and identity of participants kept confidential. All participants were treated for tungiasis according to the National Policy Guidelines on Prevention and Control of Jigger Infestations in Kenya [17]. This was by (removing the embedded flea with a sterile needle and disinfection of the skin lesion) or bathing the affected area in 0.05% potassium permanganate for 10 minutes. A nurse who was part of the team also vaccinated the participants against tetanus. Severe cases were referred to their health center by Community Health Extension Workers from the study area, who have a functioning referral system in place.

## Results

**Fig 1:**
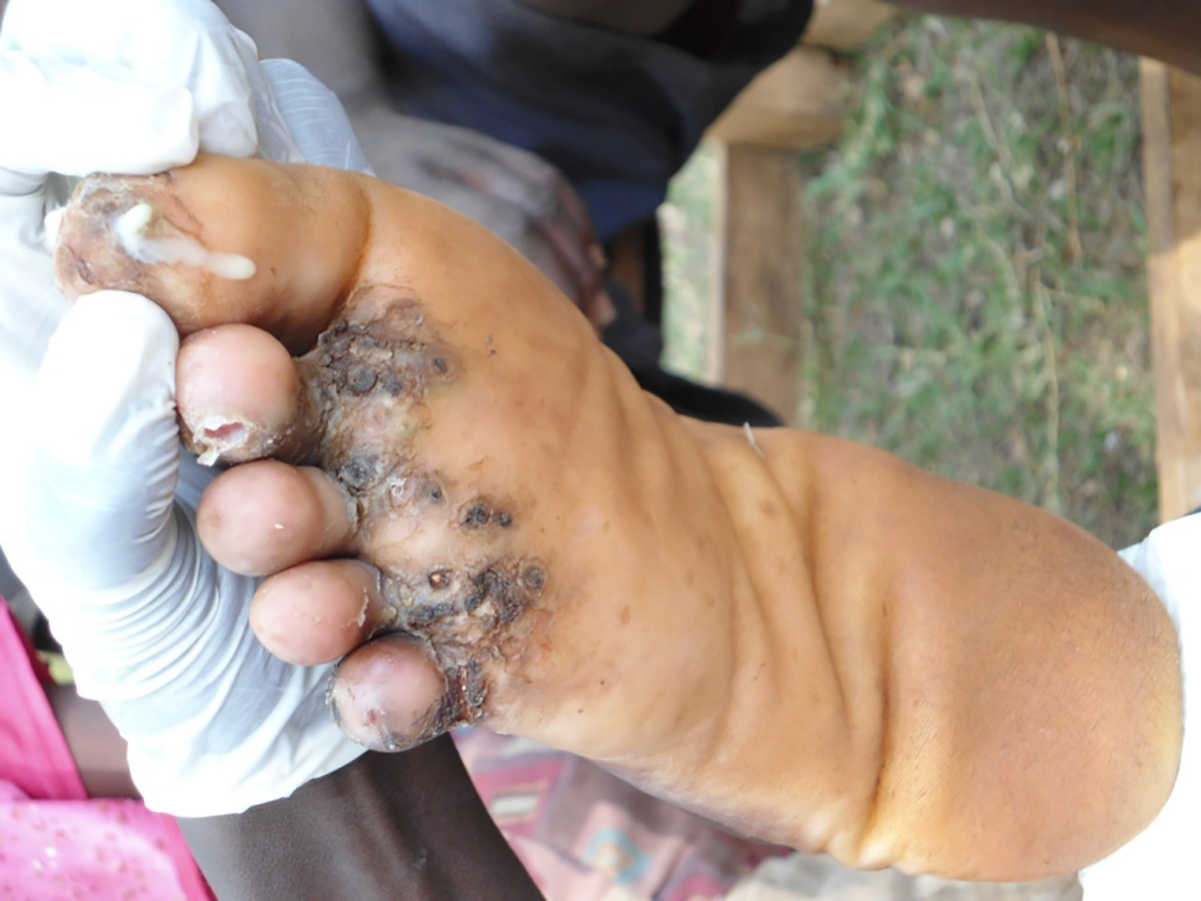
Photo of a lesion located at the base of the first toe oozing pus. One of the clinical signs of secondary infection of lesions caused by the jigger flea.

**Fig 2:**
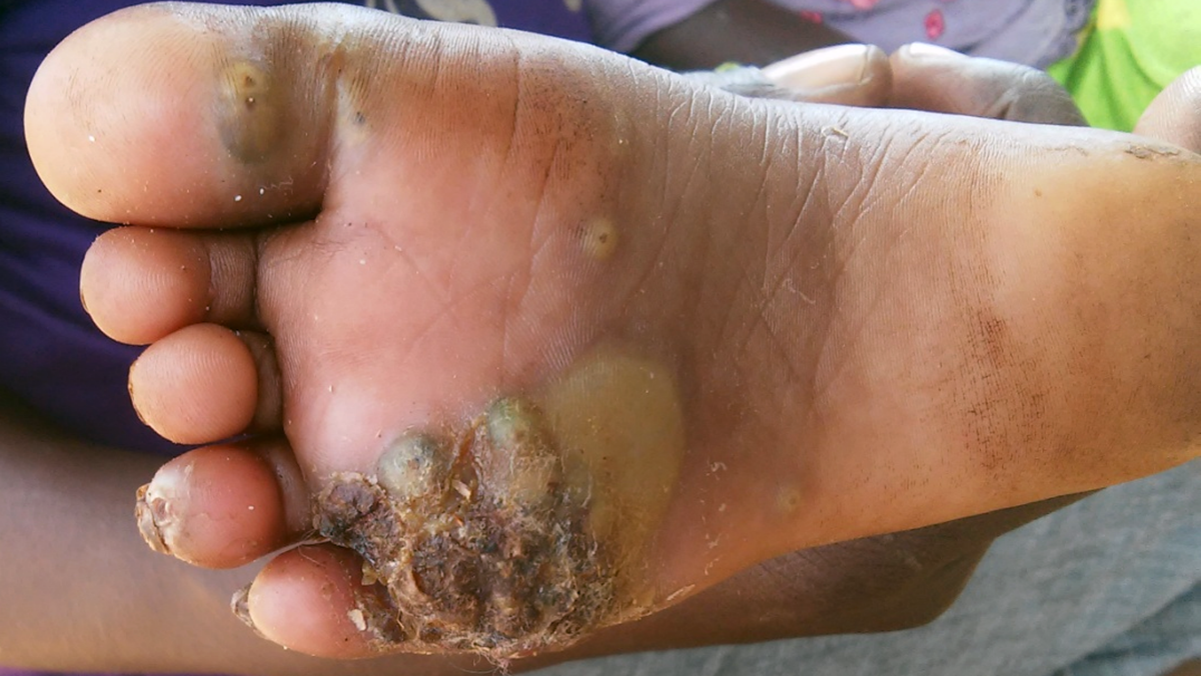
A jigger infested foot of a five year old showing clinical signs of secondary infection. Erythema and formation of pus is visibe on the upper part of the foot.

In the three sublocations from Vihiga County, 103 people were identified as having tungiasis. Swabs were taken from 37 patients who had lesions with clinical signs of secondary infection (tenderness, oedema, erythema with or without pus) Figs 1 and 2. Out of the 37 patients, from whom swabs were collected, specimen were positive in 29 and 8 had no growth. The proportion of male to female was 23 to 14 respectively. The median age was 12 years with a range of 5–80 years.

**Fig 3:**
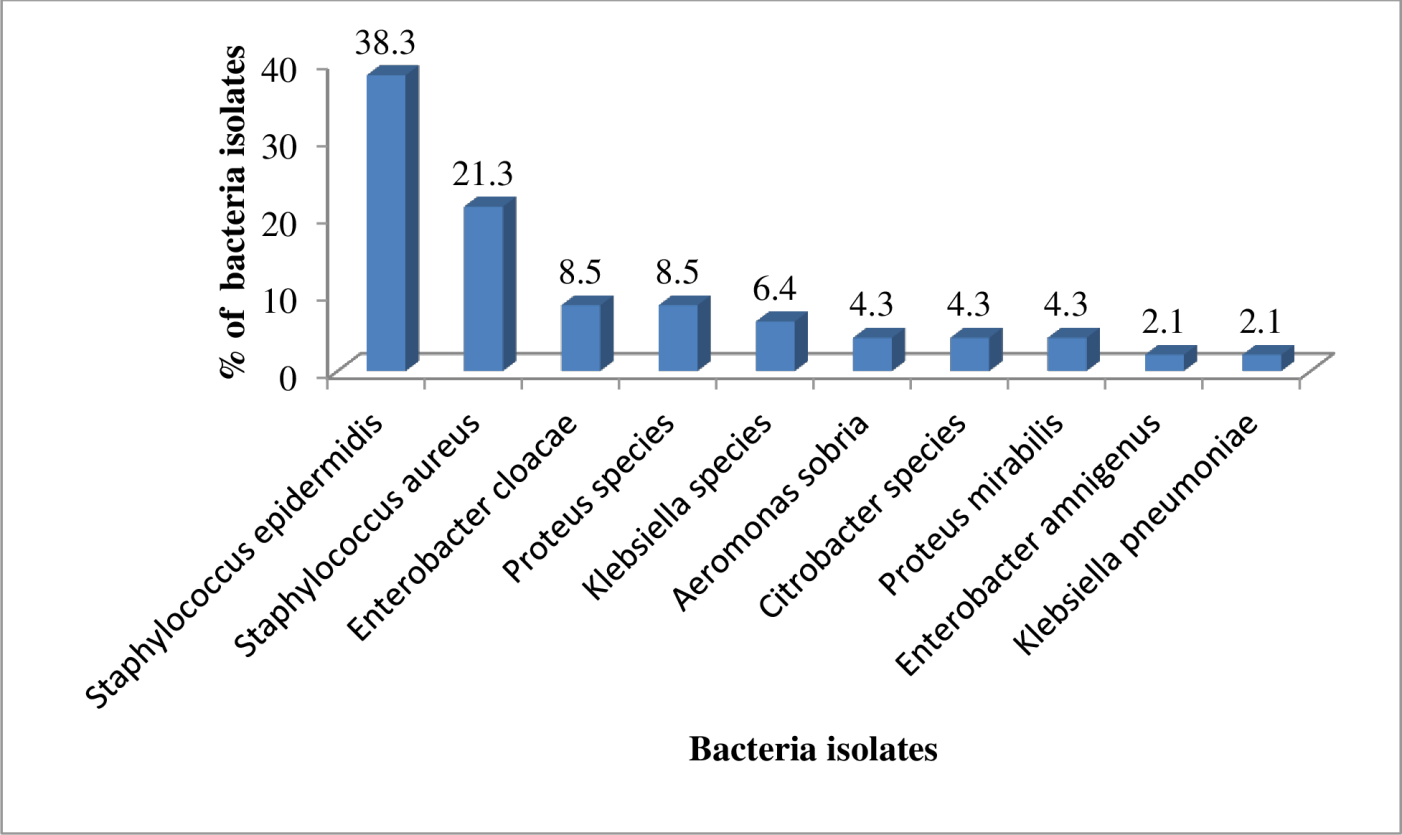
Bacteria isolated. The ten strains of bacteria isolated in this study with *S*. *epidermidis* being the highest and *K*. *pneumoniae* being the least bacteria isolated.

From the 29 Tungiasis patients up to 10 strains of bacteria were isolated. The most common being *S*. *epidermidis* (38.3%) followed by *S*. *aureus*(21.3%) and the least bacteria isolated being *K*. *pneumoniae* (2.1%). The other bacteria isolated are shown in Fig 3.

Out of the ten strains of bacteria isolated two were Gram positive [*S*. *epidermidis* (38.3%) and *S*. *aureus* (21.3%)]. Eight were Gram negative namely *Enterobacter cloacae* (8.5%), *Proteus species* (8.5%), *Klebsiella species* (6.4%), *Aeromonas sobria* (4.3%), *Citrobacter species* (4.3%), *Proteus mirabillis*(4.3%), *Enterobacter amnigenus* (2.1%) and *K*. *pneumoniae* (2.1%).

### Polymicrobial infection

Out of the 29 patients, 20 (69%) had a single strain of bacteria whereas nine patients (31%), had more than one strain of bacteria. Most of the single strain infection were Gram positive, *S*. *epidermidis* and *S*. *aureus*; 60% and 20% respectively. The rest were *Proteus species* (10%), *Klebsiella species* (5%) and *E*. *cloacae* (5%).

In the polymicrobial infection, majority 66.7% (6/9) were *S*.*aureus* and *S*. *epidermidis* (2/9) (22.2%) and their combinations as shown in Table 1. Up to 17.2% had two different types of bacteria, three and four polymicrobial infections had 3.4% each and two patients (6.9%) had five different bacteria.

**Table 1:** Polymicrobial infection isolates.

Patient	Isolate 1	Isolate 2	Isolate 3	Isolate 4	Isolate 5
1	*S*. *aureus*	*S*. *epidermidis*	none	none	none
2	*S*. *aureus*	*S*. *epidermidis*	none	none	none
3	*S*. *aureus*	*E*. *amnigenus*	none	none	none
4	*S*. *aureus*	*E*. *cloacae*	none	none	none
5	*S*. *epidermidis*	*Klebsiella species*	none	none	none
6	*S*. *aureus*	*S*. *epidermidis*	*P*. *mirabilis*	*none*	*none*
7	*S*. *epidermidis*	*E*. *cloacae*	*Klebsiella species*	*Aeromonas species*	*none*
8	*P*. *mirabilis*	*Proteus species*	*Citrobacter species*	*K*. *pneumoniae*	*Aeromonas species*
9	*S*. *aureus*	*S*. *epidermidis*	*E*. *cloacae*	*Proteus species*	*Citrobacter species*

### Bacterial drug sensitivity

Once identified, bacterial isolates were further tested for drug sensitivity to commonly prescribed drugs using the Kirby Bauer disk diffusion method. Eleven drugs were used to test susceptibility of the Gram negative isolates.All the isolates were sensitive to ciprofloxacin, cefotaxime, norfloxacin, gentamicin, nalidixic acid, chloramphenicol (Table 2). Ampicillin had the highest resistance of 52.6%. All the Gram negative bacterial isolates were resistant to atleast one or more drugs except *E*. *amnigenus*.

**Table 2:** Susceptibility profiles of Gram negative bacteria.

	% of bacteria resistant to the drug											
Isolates		Cip	CTX	NOR	CN	NA	C	AMP	AMC	TE	CAZ	CXM
*A*. *sobria*	n = 2	0%	0%	0%	0%	0%	0%	**50.0%**	0%	0%	0%	0%
*Citrobacter species*	n = 2	0%	0%	0%	0%	0%	0%	**100.0%**	**100.0%**	0%	0%	**50.0%**
*E*.*amnigenus*	n = 1	0%	0%	0%	0%	0%	0%	0%	0%	0%	0%	0%
*E*. *cloacae*	n = 4	0%	0%	0%	0%	0%	0%	**75.0%**	**25.0%**	**50.0%**	**25.0%**	**25.0%**
*K*.*pneumonia*	n = 1	0%	0%	0%	0%	0%	0%	**100.0%**	0%	0%	0%	0%
*Klebsiella species*	n = 3	0%	0%	0%	0%	0%	0%	**100.0%**	0%	0%	0%	0%
*P*. *mirabilis*	n = 2	0%	0%	0%	0%	0%	0%	0%	0%	50.0%	0%	0%
*Proteus species*	n = 4	0%	0%	0%	0%	0%	0%	0%	0%	**75.0%**	0%	0%

Cip = ciprofloxacin, CTX = cefotaxime, NOR = norfloxacin, CN = gentamicin,NA = nalidixic acid, C = chloramphenicol, AMP = ampicillin, AMC = amoxicillin clavulanic acid, TE = tetracycline, CAZ = ceftazidime, CXM = cefuroxime

*Enterobacter cloacae* showed resistance to ampicillin (75.0%), amoxicillin clavulanic acid (25.0%) tetracycline (50.0%), ceftazidime (25.0%) and cefuroxime (25.0%). *Citrobacter species* showed resistance to ampicillin (100.0%), amoxicillin clavulanic acid (100.0%) and cefuroxime (50.0%). The two proteus species had resistance to tetracycline; 50.0% and 75.0% respectively (Table 2).

All the Gram positive were sensitive to norfloxacin, ofloxacin, meropenem and gentamicin drugs. However they were resistant to clindamycin, kanamycin, oxacillin, erythromycin, nalidixic acid, trimethorprim sulfamethoxazole, chloramphenicol and tetracycline (Table 3). nalidixic acid had the highest bacterial resistance (31.0%) followed by clindamycin (20.7%).Three patients (10.3%) had *S*. *aureus* isolates that were methicillin resistant (MRSA).

**Table 3:** Susceptibility profiles of Gram positive bacteria.

		Drugs											
Isolate		NOR	OFX	MEM	CN	DA	K	OX	E	NA	SXT	C	TE
S. aureus	n = 10	0%	0%	0%	0%	30.0%	10.0%	20.0%	10.0%	50.0%	10.0%	0%	20.0%
S. epidermidis	n = 19	0%	0%	0%	0%	15.8%	0%	5.3%	5.3%	21.1%	5.3%	5.3%	5.3%

NOR = norfloxacin, OFX = ofloxacin, MEM = meropenem, CN = gentamicin, DA = clindamycin, K = kanamycin, OX = oxacillin, E = erythromycin, NA = nalidixic acid, SXT = trimethorprim sulfamethoxazole, C = chloramphenicol, TE = tetracycline

Both Gram negative and Gram positive bacteria isolates were sensitive to Gentamicin and Norfloxacin drugs.

## Discussion

Secondary bacteria infection of the lesions caused by jiggers remains a commmon occurrence among communities affected by this parasite [18]. This may be attributed to the fact that the flea interferes with the integrity of the skin which is the first defence of the body against microbes [9]. There is paucity in the number of bacteriological studies that have been done to investigate secondary infection of the lesions in tungiasis patients [7]. In this study, ten different strains of aerobic bacteria were isolated, eight of which were Gram negative and two were Gram positive. These findings corroborate a previous study carried out in Brazil Feldmeier et al., 2002 [7] which reported isolation of aerobic bacteria from tungiasis lesions. However in that study *Streptococcus pyogenes*, *Streptococci serogroup G*, *Enterococcus faecalis*, *Morganella morganii*, *Pseudomonas species* and *Bacillus species* were among the aerobic bacteria species isolated but missing in the present study. In this study, *Aeromonas sobria*, *Citrobacter species* and *Enterobacter amnigenus* were isolated for the first time. This discrepancy may be attributed to the different geographical regions and environmental factors.

Other than aerobic bacteria, anaerobic bacteria like *Peptostreptococcus species*, *Clostridium bifermentans and Clostridium sordelli* have also been reported [7]. *Clostridium tetani* bacteria which causes tetanus has been implicated in some patients presenting with tungiasis [19, 11].

Lesions caused by *Tunga penetrans* can be infected with several bacteria strains at the same time. In the present study, polymicrobial infection was observed in 31% of the patients assessed with 6.9% of these having up to 5 different bacteria strains. These results are consistent with a previous study which reported a single patient having up to 5 different pathogens [7]. Implying that antimicrobial sussceptibility testing on bacterial isolates from tungiasis patients would guide in identification of appropriate antibiotics and treatment therapy in these patients.

The Gram negative bacteria isolated in this study were found to be sensitive to ciprofloxacin, cefotaxime, norfloxacin, gentamicin and nalidixic acid drugs while the Gram positive were sensitive to norfloxacin, ofloxacin, meropenem and gentamicin. Norfloxacin is a broad spectrum fluoroquinoline that is active against both Gram negative and Gram positive bacteria. Gentamicin is an aminoglycoside that is effective against a wide range of pathogens as well. Several studies including the present study confirms their considerable anti—bacterial activities [20,21,22].

In endemic communities, tungiasis is not recognised as a disease that warrants medical attention. In other communities it’s thought to be a curse or witchcraft hence the affected people rarely seek or get treatment instead they wait to die [23]. The public Should be advised that it’s a parasitic infection that can be managed and if left unmanaged can be life threathening due to secondary infections. In the event that bacteria gets into the blood stream, it could lead to systemic infections which can be fatal [24,25].

Previously, some of the bacteria isolated in this study were considered to be of low pathogenicity. However with increase in knowledge and updated technology, this school of thoght is rapidly being set aside due to the new evidence available. For instance, coagulase negative Staphylococci which were thought to be harmless commensals, are now more than ever considered pathogens of medical importance causing considerable infections of the blood stream and other internal organs once they become invasive [26, 27].

The challenges posed by drug resistance compounds the problem even more. Treatment outcomes of resistant bacteria like methicillin resistant *S*. *aureus* (MRSA) infections are worse compared to the sensitive strains as it’s associated with increased morbidity and mortality [28]. MRSA used to be associated with hospital acquired infections but is now being isolated in a broad spectrum of community acquired diseases [29, 30]. Further, most MRSA strains are also found to be multi drug resistant [28]. This was observed in this study, since the MRSA isolated were also resistant to clindamycin, kanamycin, erythromycin, nalidixic acid, trimethorprim sulfamethoxazole and tetracycline.

Several studies have shown that there is an association between the use of antibiotics in livestock reared for food production and emergence of antibiotic resistance in human beings [31, 32, 33, 34]. Other aspects implicated for spread of antibiotic drug resistance are, poor hygiene, contaminated food, polluted water, overcrowding and compromised immunity due to malnutrition or HIV. In addition, we also have misuse and consumption of sub optimal doses of antibiotics hence inducing selection pressure for antibiotic resistance [35].

One limitation of the study is that we were not able to determine if the tungiaisis patients were taking any antibiotic medication prior to participation in the study or their immunity status. These aspects may have further explained whether they had any role in the observed antibiotic resistance. Therefore there is a need to carry out a follow up study to focus on the cause of antimicrobial drug resistance among tungiasis patients. The study was not able to isolate anaerobic bacteria due to lack of equipment at the time.

### Conclusion

The findings from this study confirm the presence of resistant bacteria in tungiasis lesions hence highlighting the significance of secondary infection of the lesions in endemic communties. This therefore implies that the treatment regimen for tungiasis especially in severe cases should be expanded to include antibiotics. Antimicrobial susceptibility testing may be considered to guide in identification of appropriate antibiotics. Norfloxacin and gentamicin have shown to be very effective against both Gram negative and Gram positive bacteria. In severe tungiasis where sepsis is observed, a broad spectrum drug may be considered at the onset to avoid delay in starting treatment as results from cultures are awaited.
